# Covid-19, Trends in Global Mission, and Participation in Faithful Witness

**DOI:** 10.1177/0265378820970225

**Published:** 2020-10

**Authors:** Paul Bendor-Samuel

**Affiliations:** Oxford Centre for Mission Studies, UK

**Keywords:** world Christianity, Christendom, mission paradigm, mission, identity

## Abstract

Mission is shaped by the life and experience of the church, both past and present, and this in turn is the function of both the work of the Spirit of God and the interaction of the people of God with their contexts. In line with this position, we examine the impact of Covid-19, highlighting some elements of the global context of mission, trends in world Christianity and mission. We then explore how global mission is in a process of realignment that has the potential to be enhanced through embracing the conditions Covid-19 has imposed on us. Finally, we consider the need for deep reflection on our identity if we are to take the opportunity to bear faithful witness in this moment.

## Introduction

We are living in a time of major disruption, not least in global mission. The model inherited from European Christendom is being challenged in profound ways. While it is still early to speak definitely, we sense that the Covid-19 pandemic is primarily acting as an amplifier of what is already happening rather than introducing something fundamentally new. Nonetheless, in bringing certain realities into sharp focus, the church is being gifted with an opportunity to reexamine some of our most basic assumptions about how we participate in the mission of God. The pandemic has stimulated enormous local activity by Christians as well as putting a brake on some aspects of mission, particularly those related to mission as sending. The current global crisis highlights the action of the Spirt of God in our world fractured by disease and suffering as well as injustice and inequalities, so often multiplied by human choice and action.

The Spirit works in mysterious ways. We know from theoretical works that while crises like Covid-19 lead to much suffering, they can also lead to religious change and transformation^1^. In this sense, the locations of crises can be opportunities for ministry and mission. This is an opportunity to take stock and envision global mission in ways that are, perhaps, more appropriate for this moment in history.

The first part of this paper, therefore, begins with a broad perspective on the global context of mission, trends in world Christianity and mission. Here Covid-19 pandemic is highlighted as a particular crisis facing humanity.

## Global Context of Mission

That we talk about trends at all is an indication that ours is a globalized generation, aware of the big picture and able, to some degree, to reflect in global perspective. Thinking of trends can be helpful as long as it does not obscure the reality that the local is always “exceptional.” There is a tension here: we seek to understand the global while at the same time learning to listen and pay attention to the uniqueness of the local. While the Covid-19 pandemic has been global in extent, its impact and response have been experienced in widely differing ways that make the pandemic a profoundly local phenomenon.

It is important to hold this global–local tension if we are to avoid unhelpful confidence in analysis that can lead to a fixation with strategy, which in turn undermines dependence on God and sensitive listening to the Spirit and the local context. At this moment, the world population stands at 7.8 billion people, of whom more than 50% are urban, middle class and older than 30^2^. An aging^3^, middle class,^4^ and urban population^5^ are trends but such data masks huge diversity in national and local contexts.

In terms of global issues, it is suggested that Covid-19 is but one of three major challenges dominating the landscape. Reflecting on mission, on what it means to bear faithful witness today, we need to recognize all three, since they are inextricably interwoven and mutually reinforcing in their impacts.

### Covid-19 and Poverty

We have entered an era of pandemics—some new (SARS, MERS, Influenza H5N1, Swine flu, Ebola, Zika), while others are long known diseases like malaria, yellow fever, measles, and dengue. The uncertainty and restrictions caused by Covid-19 are likely to be with us for the next two to three years, but it will not be the last novel virus to cause disruption. Most new epidemics have been zoonotic, that is, caused by a virus jump from an animal host. This is likely to continue and probably increase.^6^ Covid-19 has been unusual in its spread and scale of disruption caused both by the disease and by attempts to contain it. Covid-19 is intensifying many of the major global issues threatening communities, including extreme poverty, the environmental impact of climate change, food and water insecurity, and gender violence.

Between 1800 and 2017, extreme global poverty fell from 85% to 9%, with the biggest drop from 50% to current levels happening since 1966.^7^ Much of the drop took place due to changes in China’s increasingly urban population and more recently to the rapid economic growth occurring in parts of West and East Africa. At the same time, average life expectancy has risen from 31 years in 1800 to 73.2 years in 2020.^8^ Although extreme poverty has been falling, 2020 will mark the first year in decades when that trend is reversed.^9^ The situation in Africa is particularly severe, where incredible economic growth in some contexts is now in reverse. It has been estimated that those suffering from acute hunger globally could double.^10^ For fragile states and communities, Covid-19 is simply another body blow.

### Environmental Disaster

Human beings do not respond well to slow onset disasters.^11^ Unlike Covid-19, the climate crisis is a genuine existential threat to our world. Drought, fires, storms, flooding, rising sea levels, and the relentless extinction of species combine to threaten global food and water security and habitable space.

The huge reduction in the global economy, ground, and air travel reduced global CO_2_ has reduced global CO_2_ emissions by an estimated 7% for 2020 if some restrictions continue till the end of the year. Yet for global rise in temperature to stay under 1.5 degrees rise, we would need a similar reduction (7.6%) every year this decade.^12^

Climate change and the global economy are interconnected. Morgan Stanley estimated that 16 climate events cost the United States alone a staggering $309 billion in 2017.^13^ According to a *Financial Times* report, the then Bank of England governor, Mark Carney, warned in 2015 that “Once climate change becomes a defining issue for financial stability, it may already be too late.”^14^

Fundamental questions about the sustainability and stability of the global economy are not just driven by Covid-19 and climate change. The myth of limitless growth has been brought into sharp relief by events such as the 2008 financial crisis. As activists around the world urge their governments to “Build Back Better”^15^ following Covid-19, it is clear that simply greening the economy will not be sufficient. We desperately need a new economic model that moves us from an economy of consumption to one of needs-based, sustainable production in which all benefit.^16^

### Racism, Postcolonialism, and Neocolonialism

Global insecurities have fed the rise of populist, authoritarian regimes.^17^ This in turn connects with a third global issue intersecting with Covid-19 and the climate crisis—that of racism and the legacy of colonialism globally. The focus on systemic injustice and inequality has been heightened following George Floyd’s unlawful killing in the United States. This sparked global outrage, from Brazil to Beirut, Syria to Singapore and generated solidarity among populations who feel oppressed by dictatorship, brutal policing, and unaccountable political authorities.^18^

Forces of globalization, whether economic or cultural, have generated a counternarrative expressed in nationalism, frequently allied to religious radicalism. These forces need to be seen against a postcolonial backdrop where as recently as 1914, 85% of the earth’s landmass was controlled by European and, predominantly, British powers. One hundred years may seem a long time, but the impact of colonialism lives deep in the psyche of oppressed communities fed by continuing daily realities of inequality. Opposition to globalization, experienced as a form of neocolonialism, can be understood as a profound struggle for identity and belonging.

The comments above are a very brief commentary on three global trends that profoundly affect World Christianity and Christian mission. Space precludes any exploration of other equally important issues such as urbanization, migration, and displacement of peoples, the changing nature of economic and military power with the rise of China, corruption, the impact of Artificial Intelligence and technology allied to fundamental questions of what it means to be human.

## Trends in World Christianity

Following the comments above, it is worth pointing out that churches and Christian Non-Governmental and Faith Based organizations continue to be central players in efforts at poverty reduction, education, and health care. This is not just true of those contexts in which state actors are unable or unwilling to provide basic services. In the UK, Christian groups have been at the forefront of the hospice care movement, food banks, and community initiatives to support young mothers and infants, the care of the elderly and so on. As state provision becomes increasingly costly, the space for Christian action grows. In Muslim contexts, Christian service, provided unconditionally, remains a central way to bear faithful witness to the grace and goodness of God.

Those who identify as Christian have never reached beyond a third of the world’s population and by 2050, this is set to fall to 31%. Islam, in contrast, estimated at 26% in 2010, is set to rise to 30% by 2050. As Andrew Walls famously reminded us many years ago, Christianity, unlike Islam, is not territorial. The shift in Global Christianity from North and West to South and East is well documented. The multiplication of Christian denominations reflects these demographic shifts within World Christianity. By 1984, in Africa alone there were over 7,000 independent denominations in 43 African countries, representing new expressions of church that are unrelated to earlier mission efforts.^19^

While recognizing the shifts in geographic locus, perhaps we have been slower to recognize the extraordinary growth in the diversity of the church in the past 50 years. As recently as the 1990s, there were numerous countries, including many majority Muslim nations in North Africa, the Middle East, and Central Asia, in which there was no known indigenous church. Today we can rejoice in vibrant communities of Christ followers in every nation. We will pick this up below but suffice it to say here that significant movements toward Christ have been documented from within Islam, Hinduism, various strands of Buddhism, and ideological contexts of communism. Only secular materialism appears resistant to significant church growth.

The church has not only become more diverse through multiple movements to Christ within particular communities. Diversification has occurred through people on the move, whether that be those from Sumatran Muslim tribal groups finding Christ in Jakarta mega-churches, Afghan migrants in Germany, or Christians on the move such as Sudanese refugees in Cairo, Filipino maids in Hong Kong or Nigerian business people in London. The UN estimates that at the end of 2019 there were 79.5 million displaced people, 85% of whom were hosted in developing world countries.^20^ This does not include the millions who have migrated for economic reasons, studies, or family connections. While we have tended to describe the church by geography, ethnicity, or religious background, these categories are increasingly inadequate descriptors in today’s world of kaleidoscopic movement. More than at any point in history, the church is faced with the opportunity to demonstrate the sign of the Kingdom through a united people sharing a common identity in Christ. Given the earlier comments about racism and postcolonialism, this sign is profoundly needed.

Two other trends are worth mentioning briefly. First, we note the increasing persecution and the marginalization of Christianity. Open Doors, in their 2019 report noted a number of major trends shaping persecution of Christians:

- Authoritarian states are clamping down and using legal regulations to control religion. Examples include China, North Korea, and Vietnam.
- Ultranationalists are depicting Christians as “alien” or “western” and trying to drive them out. Here examples include parts of India, Myanmar, Turkey, Nepal, and Bhutan.
- Radical Islam has moved from the Middle East to sub-Saharan Africa and seen in armed insurgencies in Nigeria, Mali, Burkina Faso, Chad, and Niger as well as Somalia, Yemen, and Libya.^21^

If persecution reflects attack from outside, we should also note internal factors that render the church vulnerable from within. The number of adherents is no measure of strength, as demonstrated by those contexts where 25, 40, or even 80% of the population profess Christian faith and yet society does not remotely reflect the Kingdom of God. Kenyan scholar George Kinoti laid the blame at the feet of Western missionaries who brought a “spiritual” gospel that failed to integrate discipleship with every aspect of communal living and society.^22^ There was a reduction of the gospel of the Kingdom in ways that separate the kingdom from the King. Kinoti does not blame those missionaries but points out that they failed to recognize how their worldview was shaped by their own culture. This is not unique to western mission in East Africa. The global church consistently underestimates the degree to which context shapes us, resulting in failure to develop biblically authentic, contextualized discipleship. Like woodworm that can eat the heart from a mighty tree, leaving it vulnerable to sudden and catastrophic collapse in storms, superficial expressions of discipleship are always deceptive. We may think the church is healthy due to size when it is in fact vulnerable to rapid decline due to an inability to reflect theologically and act courageously in contextually appropriate ways. The North African church is an example of an apparently large church going into catastrophic decline and most assessments today suggest internal factors as the cause rather than simply Islamic conquest.^23^

With this brief overview of trends in global context and World Christianity, we now consider how these are reflected in world mission.

## Trends in Mission

### The Prevailing Cross-cultural Mission Paradigm

The prevailing mission paradigm is under pressure and Covid-19 is accentuating the fractures that have been there for some time. Within Protestant expressions of church, the idea that mission is centred on sending developed in the late 18th century, flourished in the 19th and came under increasing pressure in the 20th. However, Dana Roberts comments, “By the end of the 20th century the most significant development in the structure of missions was not the end of the missionary movement but its transformation into a multi-cultural, multifaceted network.”^24^ Mission sending had moved from “West to rest” to “from everywhere to everywhere.” Newer mission movements are now contributing tens of thousands of workers from Latin America, Africa and parts of Asia, notably South Korea and, more recently, the Philippines.

This movement has resulted in the formation of hundreds of new mission-sending agencies as well as the internationalization of historic agencies who now generally function as multicultural organizations and teams. Newer expressions of sending are described as “reverse mission,” with those from the Global South and East moving to evangelize the post-Christian West.^25^ As mission agencies have struggled for legitimacy in a global context, larger churches, mega-churches, and networks have sent workers directly.

These realities might suggest that the modern mission paradigm, focused on sending and the cross-cultural missionary, is alive and well. While some hail these innovations as a new era of mission, we suggest these developments simply reflect modifications to the existing paradigm. This understanding of mission is faltering for numerous reasons, including:

- Unsustainable financial systems in both old and newer sending contexts.
- The proliferation of mission agencies and Christian nongovernmental organizations too often doing their own thing without relationship to the Body of Christ locally.
- Visa restrictions arising from a range of circumstances, including suspicion of outsiders and the existence of locally trained professionals.
- An emphasis on short-term sending. Long-term is reduced to a few years, resulting in decreased cross-cultural ministry preparation, language, and cultural acquisition.

More important than these issues, mission reduced to sending is increasingly ill-adapted to today’s very varied mission contexts and is increasingly out of step with our understanding of the nature of mission. The current system has shaped mission from historic sending contexts but also the new mission movements from Latin America, Africa, and parts of Asia. It fundamentally fails to take account of the degree to which the sending model of mission reflects a Christendom view of the world.^26^ Alan and Eleanor Kreider have shown that the “sending and going” model fitted within a broader Christendom paradigm, in which four elements were tightly interwoven^27^:

- Mission defined by geography, with parts of the world “Christian” and parts “not yet Christian.”
- The current paradigm of mission still maintains the importance of geography with phrases like “the 10/40 window.” However, in some parts of the Protestant mission movement, geography has largely been substituted with ethnicity, with the focus on “people groups” coupled with anachronistic readings of the Greek term *ethnos* in the New Testament. While for many this remains compelling, increasingly this approach fails to take account of the complexities of identity and movement that characterize the global context and especially the growth of the church in all nations.
- Mission as the responsibility of the church.
- Today we recognize that mission is God’s—the missio Dei—and that we are called to participate in his mission. Nonetheless, the legacy of Christendom assumptions about mission is so strongly embedded in mission language, structures, and systems that it is possible to affirm the missio Dei and at the same time take over the work of mission, ignoring what God is already doing and focusing on our statistics, strategies, and resources.
- The goal of mission as the establishment of the church.
- When mission is the responsibility of the church then, naturally, church concerns will define the content of mission. When cross-cultural workers come from church contexts shaped by enlightenment thinking, church concerns may be reduced to “spiritual” activities and all that flows from that in terms of a dichotomized gospel in which the Kingdom is separated from the King, as mentioned above. Our understanding of the work of mission continues to suffer from a reductionist view of the nature of God’s mission.
- Special agents are required for mission.
- In 1974, the Lausanne Covenant noted that, “evangelization requires the whole church to take the whole gospel to the whole world.”^28^ Nearly 50 years later, mission continues to be promoted and practiced as something done by Christians with a special calling, among particular kinds of people, focused on gathered church activities.

It may be that the perpetuation of a Christendom view of mission, where mission is primarily based on cross-cultural sending, is the single biggest obstacle to the whole people of God taking responsibility to step out and participate in the fullness of God’s mission. Covid-19, along with the climate crisis and postcolonial context, offers an unparalleled opportunity to realign our understanding and practice of mission. This realignment will grasp two key realities.

First, the centrality of the people of God in a locality as the primary human instrument for mission in that context. The growth of the church in every nation over the past 30 years means that the primary witness to the gospel of the Kingdom is no longer dependent on individual cross-cultural servants but local worshiping communities of disciples. This is not to suggest that local churches exist in every community or ethnolinguistic group, but all these groups are accessible to near neighbors.

The call for a shift in focus from cross-cultural mission to local faithful witness is nothing new. It was Roland Allan’s challenge to the mission movement over 100 years ago, with the challenge to trust the Spirit of God at work in the new churches and let go control.^29^ Dr. Jay Matenga, Executive Director of the World Evangelical Alliance Mission Commission, has been listening to conversations of mission leaders globally through the Covid-19 pandemic and sees a number of emerging themes including transformative collaboration, whole-of-life discipleship, and technical advancements in service of mission. However, the strongest theme is the call to an indigenous future.^30^ Covid-19, far from being a frustration to the mission of God, could be just the restraint to the global mission industry we need if we are to reimagine how different parts of the Body of Christ act together to support faithful, holistic, local witness.

We should not underestimate the challenge. The legacy of Christendom mission remains deeply embedded in our churches, denominational structures, and agencies. If we are to “build back better” in mission, there is an urgent need to explore the assumptions and theological constructs that underpin over 200 years of Protestant mission. Key to discerning what God requires of us in “mission” today is the development of new language. Mission itself is a term freighted with assumptions, wrapped up in its Latin origins (missio, to send). If mission is no longer conceived primarily as physical sending and going, then the label itself must change.^31^ This is not to suggest there is no place for cross-cultural going. Nor does it deny the Biblical theme of Trinitarian sending and going which in turn is to be reflected in the people of God. Rather, we believe it is time to disengage the sending of the whole church into the world (John 20:21) from the modern mission movement that has laid exclusive claim to its interpretation. This exclusive claim continues to marginalize large sections of the Body of Christ. Just as the Covid-19 pandemic has reminded us of the locatedness of the experience and response to the pandemic, so also are we reminded that faithful witness to the Lordship of Jesus Christ over everything and everyone (Ephesians 1:9,10) is borne out principally through indigenous witness.

This bring us to a second reality that must shape the faithful witness of the church. The pandemic, impact of climate change, and heightened awareness of racial injustice are powerful reminders of the brokenness of our world and the iniquitous inequalities and injustices that define countless lives globally. We are reminded that God’s mission is more than simply the rescue of lost souls from a disintegrating planet but the renewal of all things (Rev 21:5) and healing of brokenness and alienation of all things in heaven and earth (Col 1:20). A narrow, reductionist, spiritualized understanding of mission fails to take into account the Biblical story, God’s self-revelation, and God’s call to His people to faithful witness through life, word, and transformed community from Genesis to Revelation. This moment provides a fresh invitation to the Body of Christ to join together what we have so often torn apart; being and doing, living and speaking, serving and prophetic proclaiming, abiding, and going.

## Embracing Identity

One of the obstacles to making deep change is the challenge it brings to our sense of identity. I was recently talking with a cross-cultural worker in Central Asia. He had left everything to become a church planter. He explained the struggle he felt as his role had become that of encourager and supporter to the indigenous leaders. He was undergoing a crisis of identity. Whether we believe it is theologically right or not, we cannot escape the reality that who we are is so often shaped by what we do. Disruptive times can be a gift in which we may discover anew our dependency on God in our engagement in his mission and the Biblical story provides plenty of examples of how this can be so. Take Abraham and the story recorded in Genesis chapter 20.

1 Now Abraham journeyed from there to the region of the Negev and settled between Kadesh and Shur. While he was staying in Gerar, 2 Abraham said of his wife Sarah, “She is my sister.” So Abimelech king of Gerar had Sarah brought to him.

Twenty-four years after the calling to leave country, household and family, now aged 99, the story of Abraham and Abimelech is sandwiched between a series of momentous encounters between Abraham and God. During his 99th year, God appears to Abram and changes his name, reaffirms his covenant through circumcision (ch17), appears at his tent and again promises a son, reveals his plans to judge Sodom and Gomorrah precipitating a remarkable intercessory conversation (ch18), destroys the cities on the plain (ch19), and finally gives the heir of the promise (ch21). Quite a year of encounters. Yet in the midst of them, we are reminded that Abraham is still a family of no fixed abode. Wandering in a land not his own, surrounded by those not his own, Abraham still lives with insecurity and fear. He was afraid for his life, for Sarah was beautiful and he was concerned that one stronger than he would overpower him and carry off his beautiful wife. This fear first surfaced years earlier on a visit to Egypt. Fear led to deception, “she is my sister,” and this became the protective mantra. Twenty-four years later, fear releases behaviour that has become the default.

The story unfolds with Abimelech taking Sarah but God intervening to protect both her and Abimelech. It’s a remarkable narrative for students of mission, in which the pagan Abimelech turns out to fear God more that the man of God, Abraham. However, what is of interest here is the way Abraham describes himself when confronted by Abimelech.

11 Abraham replied, “I said to myself, ‘There is surely no fear of God in this place, and they will kill me because of my wife.’ 12 Besides, she really is my sister, the daughter of my father though not of my mother; and she became my wife. 13 And when God had me wander from my father’s household, I said to her, ‘This is how you can show your love to me: Everywhere we go, say of me, “He is my brother.”‘“ (Gen 20:11–13)

“When God had me wander from my father’s house. . .”

The Hebrew is literally “when God caused me to wander from my father’s house.” It is almost as if Abraham is blaming God for his predicament, his sense of insecurity and fear. After all these years, Abraham is still struggling with his identity. How might it have been different if Abraham had embraced the wanderer identity, uncomfortable though it is? Whatever Abraham’s struggles at this point, his identity as a wander becomes embedded in Israel’s identity:

“you are to declare before the LORD your God, ‘My father was a wandering Aramean’,..” Deut 26:5)

The wanderer symbolized the insecurity and vulnerability of Israel’s origins. This is recalled by King David when he finally settles the Ark in Jerusalem, celebrating in song,

19 When they were but few in number,

few indeed, and strangers in it,

20 they wandered from nation to nation,

from one kingdom to another.

21 He allowed no one to oppress them;

for their sake he rebuked kings:

22 “Do not touch my anointed ones;

do my prophets no harm.” (1 Chronicles 16:19-22)

The wanderer identity came to signify not only vulnerability but the faithfulness of God and his ability to provide and protect in the midst of insecurity. Here is the point for us today, faced with the suffering and loss and the disruption of cherished certainties: the wanderer identity is a gift, pointing us to a place of vulnerability and dependence on God.

The wander identity, of stranger and alien in a land not our own, is picked up in the New Testament by the writer of 1Peter. In his opening greeting, he addresses “God’s elect, strangers in the world and scattered in .….” Note the juxtaposition of identity: elect and stranger. Both are true. Peter, rather than holding the wanderer identity at arm’s length, as something imposed by God, urges his readers to embrace it. “I urge you to live as aliens and strangers in the world” (1 Peter 2:11).

Insecurity and fear are powerful emotions, triggering behaviour that becomes ingrained over the years. The danger is that, rather than embrace the opportunity for change, we fall back on what we know, on that which soothes our sense of identity. Abraham’s story reminds us that fear is very rarely a healthy driver of behavior. Instead, we are invited to embrace the identity of the wanderer, an identity that is not primarily about geography but a posture of dependence, vulnerability, and daily obedience.

## Conclusion

We began by saying that mission is shaped by the life and experience of the church, both past and present. Covid-19 is particularly noteworthy in that for many around the world it represented a level of crisis this generation has not seen before. The experience of Covid-19 has been truly universal. For multitudes of others, it has multiplied the vulnerability, marginality, and suffering they already faced. For all of us it is a warning of the dire consequences that will inevitably follow if we do not take climate change seriously and radically alter the way we organize our work and relationships globally.

At the same time, we have sought to demonstrate the opportunity that this disruption provides to reimagine mission and realign the way we participate in mission as our faith is nurtured through dependent on God. Whether we are willing to do this will be shaped by many factors, not least our sense of identity as followers as those who bear faithful witness to the Lordship of Jesus Christ over everyone and everything.

## Notes

1. See, for example, sociologist Rodney Stark’s work on the impact of pandemics on the growth of the early Christian movement. Rodney Stark, *The Rise of Christianity*. Princetown University Press, 1996.

2. Ritchie H and Roser M (2019) “Age Structure,” retrieved from *OurWorldInData*, https://ourworldindata.org/age-structure (accessed 11 July 2020).

3. Vollset SE, Goren S, Yuan C-W, et al. (2020). Fertility, mortality, migration, and population scenarios for 195 countries and territories from 2017 to 2100: a forecasting analysis for the Global Burden of Disease Study. *The Lancet*, 14 July in DOI: https://doi.org/10.1016/S0140-6736(20)30677-2 accessed 15 July 2020

4. Kharas H (2017) “The Unprecedented Expansion” in *Global Economic and Development at Brookings*, February, https://www.brookings.edu/wp-content/uploads/2017/02/global_20170228_global-middle-class.pdf (accessed 17 July 2020).

5. Ritchie H (2018) “Urbanization”, *OurWorldInData*, September, https://ourworldindata.org/urbanization (accessed 17 July 2020).

6. Gibbs EPJ “Emerging zoonotic epidemics” in Vet Record, 157(22), https://veterinaryrecord.bmj.com/content/157/22/673.short (accessed 17 July 2020).

7. Rosling H (2019) *Factfulness: Ten Reasons We’re Wrong About The World - And Why Things Are Better Than You Think*. London: Sceptre.

8. “World Demographics,” 2020, https://www.worldometers.info/demographics/world-demographics/ (accessed 11 July 2020).

9. “Understanding poverty,” in *The World Bank*, 16 April 2020, https://www.worldbank.org/en/topic/poverty/overview (accessed 17 July 2020).

10. Anthem P (2020) “Risk of hunger pandemic as coronavirus set to almost double acute hunger by end of 2020”, World Food Programme Insight, 16 April. https://insight.wfp.org/covid-19-will-almost-double-people-in-acute-hunger-by-end-of-2020-59df0c4a8072 (accessed 17 July 2020).

11. See “Key definitions” 2020, *Platform on Disaster Displacement*, https://disasterdisplacement.org/the-platform/key-definitions accessed (13 July 2020).

12. Quéré Le (2020) Temporary reduction in daily global CO2 emissions, *Nature Climate Change*. 10: 647–653. https://doi.org/10.1038/s41558-020-0797-x (accessed 17 July 2020).

13. Morgan Stanley, 2018 Weathering the storm: Integrating climate resilience into real Assets Investing, 1-16, https://www.morganstanley.com/im/publication/insights/investment-insights/ii_weatheringthestorm_us.pdf (accessed 17 July 2020)

14. Figueres C and Zycher B (2020) Can we tackle both climate change and Covid-19 recovery? 7 May, https://www.ft.com/content/9e832c8a-8961-11ea-a109-483c62d17528 (accessed 17 July 2020).

15. “Covid-19 can be an historic turning point in tackling the global climate change crisis” 2020. 25 June, https://www.theccc.org.uk/2020/06/25/covid-19-can-be-an-historic-turning-point-in-tackling-the-global-climate-crisis/ (accessed 17 July 2020)

16. See, for example, Raworth K 2017 on “Doughnut Economics”, https://www.kateraworth.com/doughnut/ (accessed 13 July 2020).

17. Muis J and Immerzeel T (2017) Causes and consequences of the rise of populist radical right parties and movements in Europe. *Current Sociology* 65(1) October, 2017, p. 912.

18. Daragahi B (2020) Why the George Floyd protests went global” *Atlantic Council*, https://www.atlanticcouncil.org/blogs/new-atlanticist/george-floyd-protests-world-racism/, (accessed 13 July 2020).

19. Robert D. *Christian Mission: How Christianity became a world religion*. Oxford: Wiley-Blackwell, 2009, p.73

20. “Figures at a glance”, 2010. *UNHCR*, UK, https://www.unhcr.org/uk/figures-at-a-glance.html (accessed 17 July 2020).

21. Laury L (2019) “5 Major Trends” in OpenDoorsUSA, 16 January, https://www.opendoorsusa.org/christian-persecution/stories/5-major-trends-influence-global-persecution-christians/ accessed 17 July 2020.

22. George Kinoti, “Hope for Africa and what the Christian can do” (AISRED, Nairobi, 1994). Quoted in Phil Dow, *The School in the Clouds.* Pasadena: William Carey Library, 2003, p. 208.

23. See Daniel R *This Holy Seed*, 2nd Revised edition, Chester: Tamarisk Publications, 2010.

24. D. Robert, Christian Mission.…, p.73.

25. Israel Oluwole Olofinjana, ed., See World Christianity’s in Western Europe: Diasporic Identity, Narratives and Missiology, Oxford: Regnum Books, 2020

26. See M.W. Stroope, *Transcending the Modern Mission Tradition*, Oxford: Regnum Books, 2020.

27. A. Kreider & E. Kreider, *Worship and Mission after Christendom*, Milton Keynes: Paternoster, 2009, p. 16.

28. “The Lausanne Covenant,” *Lausanne Movement*, Clause 6, https://www.lausanne.org/content/covenant/lausanne-covenant (accessed 03/08/20).

29. Roland Allan, *Missionary Methods: St Paul’s Or Ours?*, London: Robert Scott, 1912.

30. Leader’s Review 2020, No.2, https://weamc.global/lb2020-2/ accessed 09/10/20.

31. Michael W Stroope, *Transcending mission: The eclipse of a modern tradition*, London: Apollos/IVP, 2017.

